# Dietary Vitamin D Intake in Italian Subjects: Validation of a Frequency Food Questionnaire (FFQ)

**DOI:** 10.3390/nu15132969

**Published:** 2023-06-29

**Authors:** Ranuccio Nuti, Luigi Gennari, Guido Cavati, Filippo Pirrotta, Stefano Gonnelli, Carla Caffarelli, Luciano Tei, Daniela Merlotti

**Affiliations:** 1 Department of Medicine, Surgery and Neurosciences, University of Siena, 53100 Siena, Italy; luigi.gennari@unisi.it (L.G.); guido.cavati@student.unisi.it (G.C.); filippo.pirrotta@student.unisi.it (F.P.); stefano.gonnelli@unisi.it (S.G.); carla.caffarelli@unisi.it (C.C.); 2 Italian Study Group on Metabolic Bone Disorders (GISMO), 00132 Roma, Italy; luciano.tei@libero.it; 3 Department of Medical Sciences, Azienda Ospedaliera Universitaria Senese, 53100 Siena, Italy; merlotti4@unisi.it

**Keywords:** frequency food questionnaire, vitamin D, nutrients, nutrition

## Abstract

Vitamin D plays a crucial role in calcium and phosphate metabolism, relating to bone health and preventing metabolic bone disorders such as rickets and osteomalacia. Vitamin D deficiency (serum 25-OH-D values <20 ng/mL or 50 nmol/L) is common also in Italian people; it is recommended to maintain levels above 30 ng/mL (75 nmol/L) in categories at risk. Supplementation and/or fortification with either ergocalciferol (vitamin D2) or cholecalciferol (vitamin D3) aimed to modify this condition have commonly been proposed. Studies about vitamin D intake are numerous in the literature but not adequately designed and are very often incomplete in Mediterranean Countries such as in the Italian population. On these bases, we performed a survey to validate a frequency food questionnaire (FFQ) specifically created to rapidly assess dietary vitamin D intake in Italian people. For this aim, the data of questionnaires were compared with results derived in the same population from a designed 14-day frequency food diary (FFD). Overall, a good correlation between FFQ and FFD was observed (r = 0.89, *p* < 0.001), both demonstrating a remarkably low vitamin D intake, irrespective of age and gender. Our data confirm that the vitamin D intake is very low in Italy, which likely contributes to hypovitaminosis D.

## 1. Introduction

Vitamin D plays a crucial role in calcium and phosphate metabolism, relating to bone health and preventing metabolic disorders such as rickets and osteomalacia [1].

Vitamin D3, known as cholecalciferol, is synthesized from 7-dehydrocholesterol under the influence of solar or UVB irradiation: it is firstly produced as pre-vitamin D3 that is subsequently converted into vitamin D3 [2,3]. The degree of epidermal pigmentation and the intensity of sun exposure modulate the production of cholecalciferol [4]. Vitamin D3 is also present in oily fish and cod liver oil [5]. Vitamin D2 (ergocalciferol) is produced in plants from ergosterol upon UV irradiation; although different in structure, its biologic activity is comparable to that of vitamin D3. Vitamin D3 and vitamin D2, collectively indicated as vitamin D, are well absorbed in the small intestine by simple passive diffusion and by a mechanism that involves intestinal membrane carrier proteins [6]. In blood, vitamin D is bound to the vitamin D binding protein (DBP); then, it is carried to fat and muscle, which represent the main sites of storage [7]. Both vitamin D3 and vitamin D2 are converted in the liver to 25-hydroxyvitamin D (25OHD) mainly by cytochrome P2R1 [8]. Generally, serum 25OHD is used to assess the vitamin D status in the blood. This is due to its longer half-life (3–4 weeks), as well as its greater circulating levels [9]. The serum level of the 25OHD reflects both the vitamin D produced endogenously and that obtained from foods [10].

The kidney, where 25OHD is converted to 1,25-dihydroxyvitamin D (1,25(OH)2D3) and 24,25-dihydroxyvitamin D (24,25(OH)2D3), represents the final step of vitamin D metabolism. The synthesis of 1,25(OH)2D3 is primarily regulated by serum levels of phosphate and calcium, by fibroblast growth factor 23 (FGF23), parathyroid hormone (PTH), and 1,25(OH)2D3 itself, with a mechanism of negative feedback. The active form of vitamin D, 1,25(OH)2D3, promotes the intestinal absorption of calcium and phosphate, but also increases renal calcium re-adsorption in the distal tubules. Moreover, 1,25(OH)2D3 promotes the mobilization of calcium and phosphate from bone, in concert with PTH and FGF23. However, in the past decades, evidence increasingly suggests that vitamin D may have a wider range of extraskeletal effects, which is also suggested by the wide expression of vitamin D receptors in most cell systems and by the capacity of some tissues to produce 25OHD and 1,25(OH)2D3 independently from the liver [1].

As previously indicated, sunlight exposure remains the major source (about 80%) of vitamin D for most children and adults [10,11,12], while the remaining 20% is provided by dietary intake [13,14]. However, we must consider that, on the one hand, vitamin D synthesis decreases with increasing distance from the equator and, on the other, there is often resistance to UV exposure because of risk of skin cancers, though the recommended amount of UV exposure is unlikely to be harmful for most people [15,16]. At the same time, a limited number of foods naturally contain vitamin D. These include fish such as salmon, mackerel, and herring, which have the highest natural amount of vitamin D3, likely derived from the high content of vitamin D3 in planktonic microalgae at the base of the food chain [17,18]. Conversely, beef liver, egg yolks, and cheese have small amounts of vitamin D, primarily as cholecalciferol and its metabolite 25OHD3 [19].

To date, the data regarding vitamin D intake in Italian people is very limited and poorly applicable to subjects at high risk of hypovitaminosis D, such as ageing individuals or subjects with chronic invalidating disorders. Recently, indirect information regarding vitamin D intake in Italian adults has been achieved by means of multiple-choice questions concerning the factors affecting the production, intake, absorption, and metabolism of vitamin D [20]. In this study (EVIDENCe-Q), the prevalence of severe deficiency, deficiency, and insufficiency were determined in 22%, 35.3%, and 43.3% of the study population, respectively. On these bases, we have developed a specific frequency food questionnaire (FFQ) with the aim of evaluating the daily vitamin D intake in healthy Italian resident subjects aged above 50 years.

## 2. Materials and Methods

In order to develop an FFQ able to identify the daily food intake of vitamin D, 50 healthy volunteers were interviewed about the intake of foods containing vitamin D during the previous 7 days, with the use of a specifically developed FFQ questionnaire. The inclusions criteria were age from 50 to 80 years, absence of cancer or cardiovascular, pulmonary, gastrointestinal, neurological, and renal diseases, and no gastric or bariatric surgery. The choice of the age range was related to higher risk to develop hypovitaminosis D and bone disorders in this population [21]. All the recruited subjects were correctly informed about the goal of the study, and they signed an informed consent. The subjects included in the study were 38 females (mean age 62.6 yrs ± 8.3 SD) and 12 males (mean age 65.9 yrs ± 8.4 SD) in apparent good health.

The study was approved by the Regional Ethics Committee (Regione Toscana, Sezione Area Vasta Sud Est). The enrolled subjects included physicians, nurses, and administrative staff working at Internal Medicine Unit of the University of Siena; family members and friends of the aforementioned subjects were also included in the study. All the enrolled subjects represent a rather homogeneous sample regarding their socio-economic situations and levels of education.

To determine the ability of the FFQ questionnaire to rapidly provide a correct estimate of the daily dietary vitamin D intake (to be used in the ambulatory setting), we compared the FFQ results with the information derived from the use of an appropriate frequency food diary (FFD) that recorded the daily intake of food containing vitamin D along a period of 14 days. Shorter reference intervals, such as 4-, 5-, or 7-day records, were excluded because they were not sufficiently representative of food habits [22,23,24,25]. The FFDs were distributed to the same subjects to whom the FFQs were administered. The study was conducted from May 2022 to September 2022.

The USDA National Nutrient Database and the CREA database (“Consiglio per la ricerca in agricoltura e l’analisi dell’economia agraria”) were utilized to calculate the vitamin D amount for each food [26,27].

### 2.1. Frequency Food Questionnaire (FFQ)

**Figure 1:**
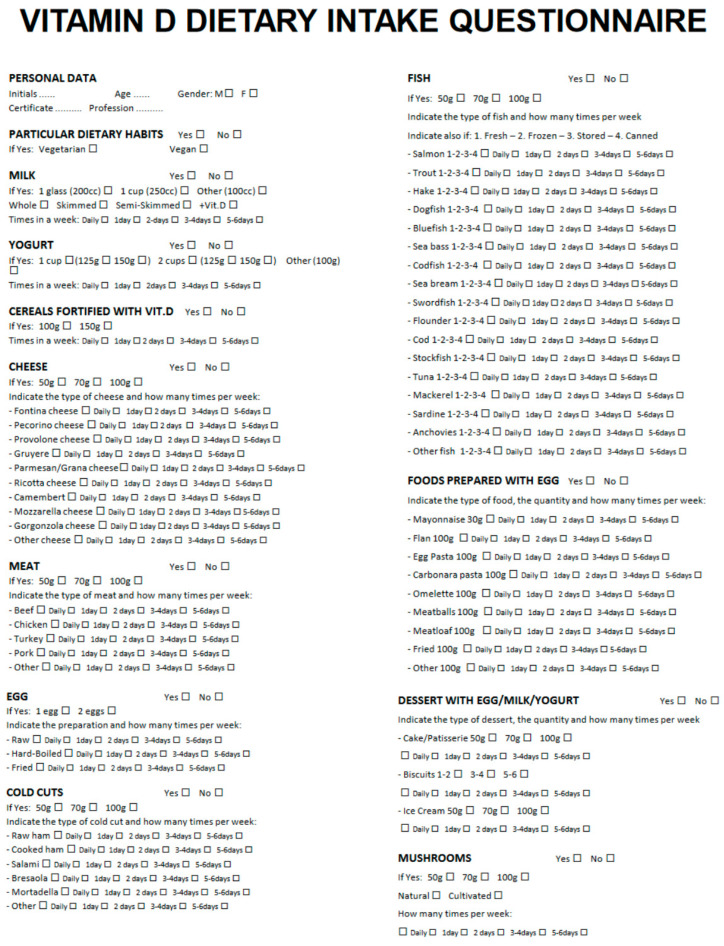
A translated English version of the food frequency questionnaire (FFQ).

A translated version of the FFQ is shown in detail in Figure 1. In its preliminary section, the FFQ reports the date of the interview, personal data of the subject (age, sex, educational degree, occupation), and possible specific eating habits (vegetarian or vegan). Subsequently, the questionnaire indicates 11 different questions, concerning the type and the amount of the intake of foods containing vitamin D. Food sources of vitamin D included: milk (whole, reduced fat, nonfat, added with vitamin D); corn flakes added with vitamin D; yogurt (whole, reduced fat, nonfat, added with vitamin D); cheese (camembert, fontina, gruyere, mozzarella, parmesan, provolone, ricotta, pecorino); meat (beef, chicken, pork, turkey, veal); fish, differentiated by fresh, frozen, dry heat, canned (bluefish, dogfish, flounder, hake, mackerel, pollock, salmon, salted cod, sardine, seabass, seabream, stockfish, swordfish, trout, tuna); egg (whole, yolk; raw, cooked); food with egg (flan, fried, omelet, mayonnaise, meatballs, pasta); cured meat (baked ham, raw ham, bresaola, mortadella, salami); desserts containing egg, milk, or yogurt (cake, ice cream, pastry, biscuits); and mushrooms. The questionnaire also provided the possibility to indicate a specific food eaten by the subject and not included in the questionnaire.

For each type of food, the amount of intake was pre-determined: regarding milk, the question was: a glass (200 mL), a cup (250 mL), or different amount; yogurt: 125 or 150 mg or other; cheese, meat, fish, cured meat, cake, and ice cream: 50 g, 70 g, or 100 g; egg: 1 or 2; and biscuits, the question regarded the number of biscuits (1/2, 3/4, 5/6). Finally, a dedicated space of the questionnaire reported how many times in a week that specific food was taken (every day, 1 time in a day, or 2/3, 3/4, or 5/6 times in a week). If the daily dose of a particular food was higher than the maximum value indicated in the questionnaire, it was specifically indicated to spread this dose over more than one day (for example, in the case of daily consumption of 200 g of cheese, this could be divided into 2 doses of 100 g for 2 days).

### 2.2. Frequency Food Diary (FFD)

The FFD, created with the aim to more accurately assess the dairy vitamin D intake during a 2-week interval in order to provide a sort of validation of the information collected via the FFQ, mainly considered the same types of foods reported in the FFQ. Thus, the subjects were invited to record every day, for a 2-week interval, each type of food containing vitamin D that they consumed and their amounts in grams (g).

### 2.3. Statistical Analysis

An analysis of variance (ANOVA) was used to compare the variances across the means of the different groups, subdivided by sex and decades of age. The relationship between mean vitamin D intakes achieved via FFQ and FFD was assessed via Pearson’s correlation coefficient. A *p*-value of less than 0.05 was considered statistically significant. Confidence intervals at 95% were also calculated.

## 3. Results

The analysis of the FFQs and FFDs was performed in 46 of the 50 recruited subjects (mean age: 63 years ± 8.5), since four diaries were excluded because information about the amounts of food were not correctly indicated.

### 3.1. Calculation of Vitamin D Intake with the FFQ

**Figure 2:**
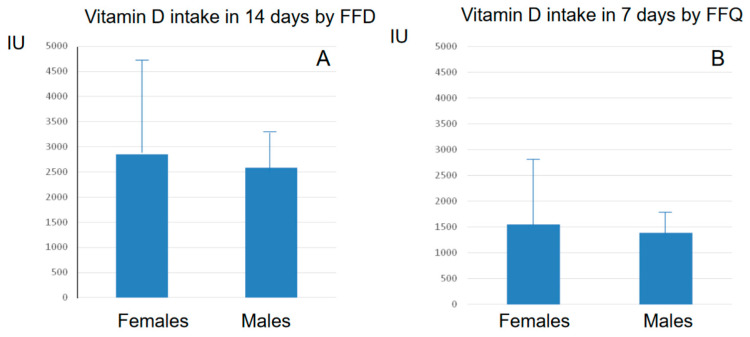
Mean (± SD) vitamin D intake in 14 days (**A**) assessed via FFD and mean (± SD) vitamin D intake in 7 days (**B**) assessed via FFQ in males and females.

**Figure 3:**
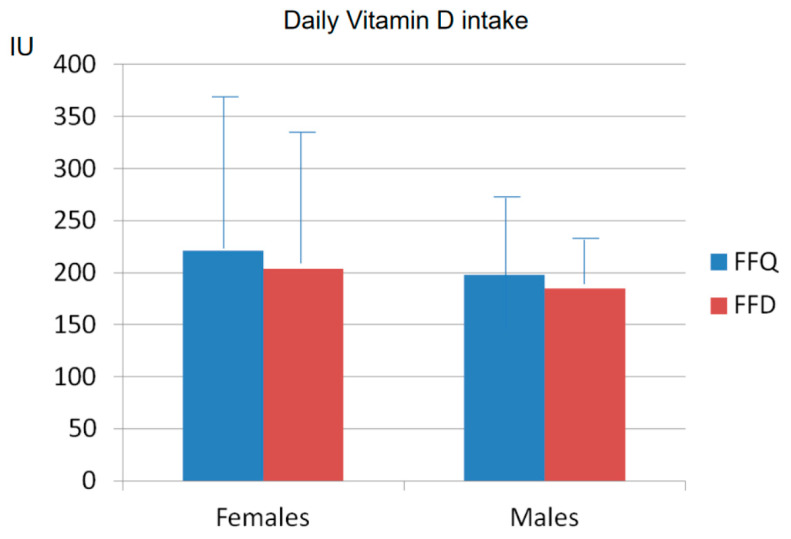
Mean ± SD daily vitamin D intakes assessed via FFQ and via FFD in females (**left**) and in males (**right**).

The analysis of the vitamin D intake, evaluated by the FFQ according to USDA and CREA databases, showed a mean weekly vitamin D intake of 1389 IU ± 408 (from 594 to 2056 IU) in the 12 males, corresponding to a mean daily intake of 198 IU ± 61). In the 34 females, the mean amount of vitamin D intake over 7 days was 1546 IU ± 999 (from 374 to 3777 IU), with a mean daily intake of 221 IU ± 143 (Figure 2 and Figure 3). These gender estimates of the vitamin D intake were thus similar and did not significantly differ.

**Figure 4:**
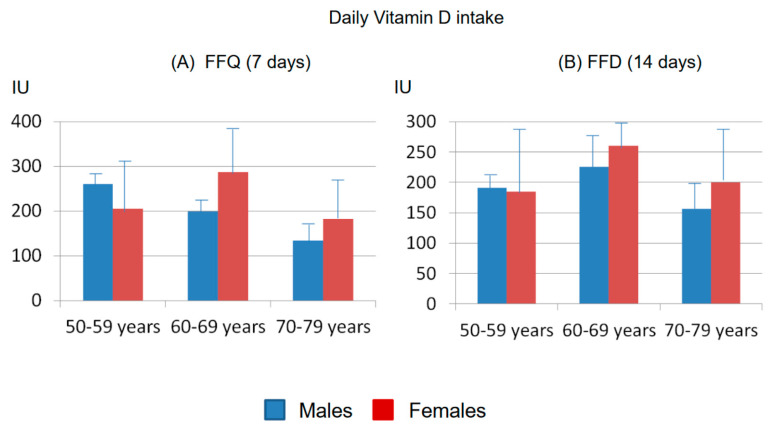
Mean ± SD daily vitamin D intakes assessed via FFQ in 7 days (**A**) and via FFD in 14 days (**B**) in males and females, sub grouped in three decades of age.

The information about vitamin D intakes in females and males achieved via FFQ were subsequently subdivided according to decades of age (50–59, 60–69, and 70–79 yrs). As shown in Figure 4 in both genders, the vitamin D intakes differed across decades: (a) 50–59 yrs: mean vitamin D intake in 7 days: 1443 IU ± 908 SD in females and 1831 ± 187 SD IU in males (mean daily vitamin D intake 206 IU ± 130 SD and 261 ± 27 SD, respectively); (b) 60–69 yrs: mean vitamin D intake in 7 days 2013 IU ± 1288 SD in females and 1389 UI ± 144 SD in males (mean daily vitamin D intake 287 IU ± 183 SD and 199 IU ± 21 SD, respectively); and (c) 70–79 yrs: mean vitamin D intake in 7 days 1277 IU ± 768 SD in females and 937 IU ± 278 SD in males (mean daily vitamin D intake 182 IU ± 109 SD and 134 IU ± 40 SD, respectively).

### 3.2. Calculation of Vitamin D Intake with the FFD

The analysis of the vitamin D intakes detected via FFDs confirmed the presence of a low vitamin D intake in both genders, as calculated over 14 days according to the USDA and CREA databases. The mean amount of vitamin D taken in 14 days was 2858 IU ± 1852 SD (from 630 to 8203 IU, mean daily intake: 204 IU ± 137 SD) in females and 2584 IU ± 652 SD (from 1370 to 4061 IU, mean daily intake: 185 IU ± 49 SD) in males, respectively (Figure 2 and Figure 3).

Moreover, in both genders, the vitamin D intakes did not significantly differ in relation to the decades of age: (a) 50–59 yrs: mean 14 days vitamin D intake: 2590 IU ± 1578 SD in females and 2667 ± 432 SD IU in males (mean daily vitamin D intake 185 IU ± 116 SD and 190 ± 26 SD, respectively); (b) 60–69 yrs: mean 14 days vitamin D intake 3646 IU ± 2494 SD in females and 2891 UI ± 759 SD in males (mean daily vitamin D intake 260 IU ± 189 SD and 206 IU ± 63 SD, respectively); and (c) 70–79 yrs: mean 14 days vitamin D intake 2439 IU ± 1613 SD in females and 2196 IU ± 555 SD in males (mean daily vitamin D intake 200 IU ± 123 SD and 156 IU ± 45 SD, respectively) (Figure 4).

### 3.3. Comparison between FFQ and FFD

From the comparison of information obtained via the FFQs and FFDs, three major points emerged.

The first point regards the questions about the amount of food intake and, consequently, the vitamin D intake. In fact, as expected, the FFDs allowed us to obtain more accurate and reliable information concerning the vitamin D intake with respect to the FFQs. In particular, the estimates of the predetermined amount of food present in the FFQs (e.g., a glass or a cup for milk; 125 or 150 g for yogurt; 50-70-100 for cheese, meat, or fish) did not completely correspond to the amount of food reported in the FFDs, in which a more accurate report about the intake of food containing vitamin D was expected.

The second point relates to the frequency of food intakes and the length of the observational survey. With respect to the 7 days of the FFQ, the 14-day period considered in the FFD obviously represented a better approach for obtaining a more realistic estimate of the dietary intake, particularly considering the limited number of foods containing vitamin D.

The third point regards the type of food. The review of the FFD reports highlighted a wider variety of foods in the various categories than those indicated in the FFQ (particularly concerning cheese and fish varieties).

**Figure 5:**
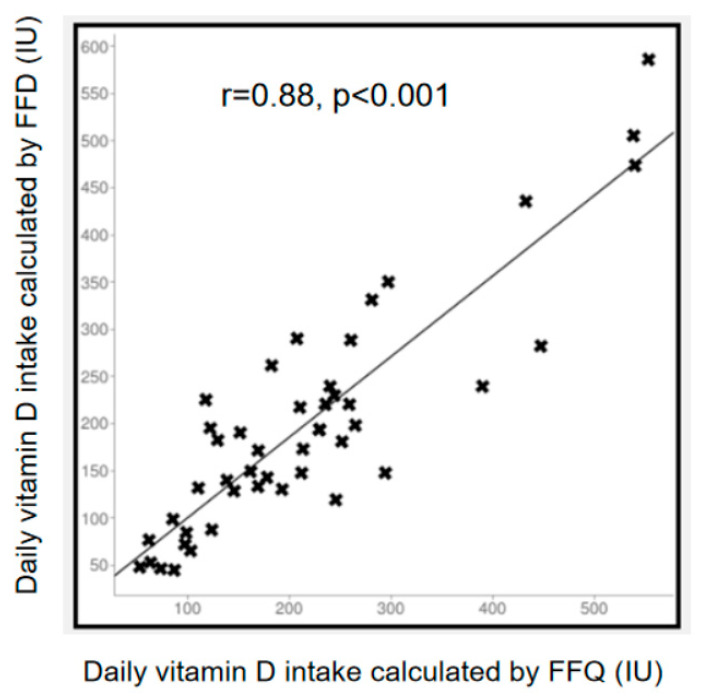
Statistically significant correlation (r = 0.89, *p* < 0.001) between the amounts of daily vitamin D intake calculated via food frequency diaries (FFDs) and food frequency questionnaires (FFQs).

Notwithstanding the above limitations, as shown in Figure 5, a higher and statistically significant correlation (r = 0.88, *p* < 0.001) was found between the daily amounts of vitamin D intakes calculated via the FFDs and FFQs.

## 4. Discussion and Conclusions

This study was performed to obtain a preliminary validation of a specific FFQ to rapidly evaluate the vitamin D intake in order to increase our knowledge about the dietary vitamin D intake in Italy. To this aim, information derived from the FFQs was compared with the results obtained in the same population with the use of a more detailed 14-day FFD. A significant and more than acceptable correlation was appreciated between the data obtained by the two approaches despite the different lengths of the observational periods and the differences concerning the precision in reporting the food amount and the kinds of food. Consequently, regarding the methodology and the items of the developed FFQ, even they can be potentially improved though they appear correct; thus, the FFQ may be considered a valid tool to investigate the dietary vitamin D intake in the outpatient setting.

Importantly, the information obtained from the FFQ and the FFD diaries allows us to underline that the vitamin D intake in the Italian, healthy population is low and does not cover the RDA in the absence of adequate sun exposure. The latter cannot be achieved at the latitudes in Italy during the winter season. Moreover, the low vitamin D intake is quite similar in females and males, and it is not significantly influenced by age. Thus, it is likely that such a low dietary intake of vitamin D contributes to hypovitaminosis D in Italy.

Other similar data from different countries indicated that the total vitamin D intake ranges from 3 μg to 5.9 μg per day [28,29,30], which is dramatically far from the 15 μg per day RDA, as recommended by the USA Institute of Medicine. Indeed, the US data, which derived from the National Health and Nutrition Examination Survey 2011–2014 that was performed on a large population sample (*n* = 16,180), indicated that the mean intake of vitamin D from food and beverage sources is around 4.9 μg/day [31].

Of interest, in this sample, circulating 25OHD levels were the lowest in the group of subjects with lower vitamin D intake (from 0 to 2.0 μg/day). Similar estimates were subsequently reported in 2015 by the Canadian Health Measures Survey (*n* = 11,992), showing mean intakes of vitamin D of 5.1 and 4.2 μg/day for males and females, respectively [32]. However, both these estimates of the vitamin D intake did not consider the contribution from dietary supplements containing vitamin D. Lower intakes have been reported in some European Countries, particularly in those where food fortification with vitamin D is limited. The UK National Diet and Nutrition Survey data collected in 2012/13 and 2013/14 showed mean dietary intakes of 3.1 and 2.5 μg/d in adult men and women, respectively, and suggested that 22% of men and 15% of women aged 19–64 years had total 25OHD concentrations below 25 nmol/L [33], reflecting a condition of severe vitamin D deficiency [34]. A more recent review by Cashman et al. indicated that only countries from Northern Europe have median vitamin D intakes above 5 mcg/day, which is likely related to the use of fortified foods; however, in the other European countries, especially in the Mediterranean area, the intake is always below 4 mcg/day and, indeed, the the European combined median value is also quite low in both genders (3.3 mcg/day in males and 2.7 mcg/day in females, respectively) [35]. The information about the vitamin D intake in Italy presented in the latter report was derived from a survey of the Italian food consumption patterns over a 7-day period in the 90 s and indicated slightly higher mean daily vitamin D intake levels than observed with our FFQ and FFD [36,37]. Additional information regarding vitamin D intake in Italy is in part available only for children and teenagers, as reported by Age.na.s (Agenzia Nazionale per i Servizi Sanitari) [38,39,40], but not in the older population. Therefore, the information released in the present preliminary survey is of particular relevance to draw attention to the problem of hypovitaminosis D and the inadequacy of nutrition as a source of vitamin D in Italy.

Indeed, hypovitaminosis D is common in Italy, as recently confirmed by cohort studies in the general population as well as in patients with metabolic bone disorders [41]. In this respect, a condition of vitamin D deficiency in the Italian population was recently defined for serum 25OHD values <20 ng/mL (50 nmol/L); however, maintaining levels above 30 ng/mL (75 nmol/L) in risk categories, as well as in patients under treatment with antiresorptive or bone osteo-anabolic drugs, has been recommended [42,43]. The consequences of vitamin D deficiency mainly involve bone and muscle, with an increased risk of secondary hyperparathyroidism, fragility fractures, osteomalacia, and enhanced risk of falls, especially in elderly subjects [44]. Likewise, adequate concentrations of 25OHD are necessary for maximizing the efficacy of anti-osteoporotic drugs [45]. Moreover, several acute and chronic disorders including autoimmune diseases, infectious and cardiovascular diseases, cancer, and diabetes have been often associated with an inadequate vitamin D status [46,47,48,49]. In keeping with the above observations, the Attica study, performed in a Mediterranean country such as Greece using a validated semi-quantitative food frequency questionnaire, indicated an inverse association between vitamin D intake and cardiovascular disease [50]. Likewise, the dietary vitamin D intake was positively associated with cognitive performance in elderly subjects from the National Health and Nutrition Examination Survey (NHANES), 2011–2014 [51]. Consequently, the opportunity of fortification strategies has been recommended as a public health policy to support the attainment of dietary vitamin D recommendations in most countries [52]. In this respect, Lamberg-Allardt et al. [53] underlined that current dietary intake recommendations are too low to maintain an optimal vitamin D status especially when UVB radiation is reduced or not sufficient and suggests that daily vitamin D intake should be increased to at least 10 µg per day and 25 µg in the elderly. Recently, the European Society for Clinical and Economic Aspects of Osteoporosis, Osteoarthritis and Musculoskeletal Diseases (ESCEO) working group analyzed the meta-analyses of randomized controlled trials on the effects of vitamin D on fracture risk, falls, or osteoarthritis, and confirmed that 1000 IU daily should be recommended in patients at increased risk of vitamin D deficiency [54].

Undoubtedly, this preliminary survey is characterized by some biases: the number of subjects included in the study is limited, not homogeneously subdivided by gender, and is representative of only a restricted area in central Italy. In any case, these results represent the first information collected in an adult Italian population concerning the daily intake of vitamin D, and they may be considered indicative of specific eating habits. As previously reported, sunlight exposure remains the major source of vitamin D, particularly at latitudes below 37 degrees north or below 37 degrees south of the equator, where the role of food intake plays a modest role in the achievement of a normal vitamin D status. Indeed, mushrooms, eggs, and oily fish may potentially contain high concentrations of vitamin D; however, these foods are not eaten in adequate amounts to have a significant impact on the vitamin D status [55,56]. Similarly, meat, milk, and cheese are not sufficiently eaten to reach the dietary vitamin D level recommended by the International Health Institutes. In Italy, the diffuse dietary habits of the Mediterranean Diet (mainly including olive oil, wheat, vegetables, and grapes), together with the lack of consistent food fortification, undoubtedly contribute to a low vitamin D intake.

Indeed, FFQs are a practical tool for the measurement of usual food consumption patterns in large surveys and, thus, are widely used because of their relatively low cost, and for the fact that they allow for easily collecting semi-quantitative data. However, they need to be validated when compared against more detailed and accurate methods of assessment [57].

In conclusion we confirmed that, in our sample of subjects from central Italy, there is an inadequate dietary intake of vitamin D and that our FFQ is able to rapidly estimate the vitamin D intake. The availability of this validated FFQ will thus allow us to replicate the study in a larger sample. Should these data be replicated in a large and representative sample of the Italian population, they will demonstrate that, in Italy, the vitamin D intake is very low and markedly contributes to hypovitaminosis D. Consequently, specific national health policies could be designed and standardized in order to correct this condition and to likely improve bone health in the general population and particularly in specific categories, where vitamin D deficiency is common.

## Data Availability

The data presented in this study are available on request from the corresponding author. The data are not publicly available due to privacy.

## References

[1] Bouillon R., Carmeliet G. (2018). Vitamin D insufficiency: Definition, diagnosis and management. Best Pract. Res. Clin. Endocrinol. Metab..

[2] Hossein-nezhad A., Holick M.F. (2013). Vitamin D for health: A global perspective. Mayo Clin. Proc..

[3] Holick M.F. (2007). Vitamin D deficiency. N. Engl. J. Med..

[4] Wacker M., Holick M.F. (2013). Sunlight and vitamin D: A global perspective for health. Dermato-Endocrinol..

[5] Webb A.R., Kline L., Holick M.F. (1988). Influence of season and latitude on the cutaneous synthesis of vitamin D3: Exposure to winter sunlight in Boston and Edmonton will not promote vitamin D3 synthesis in human skin. J. Clin. Endocrinol. Metab..

[6] Thomas K.K., Lloyd-Jones D.H., Thadhani R.I., Shaw A.C., Deraska D.J., Kitch B.T., Vamvakas E.C., Dick I.M., Prince R.L., Finkelstein J.S. (1998). Hypovitaminosis D in medical inpatients. N. Engl. J. Med..

[7] Bikle D.D., Adams J.S., Christakos S., Bilezikian John P. (2019). Vitamin D: Production, Metabolism, Action, and Clinical Requirements. Primer on the Metabolic Bone Diseases and Disorders of Mineral Metabolism.

[8] Thacher T.D., Levine M.A. (2017). CYP2R1 mutations causing vitamin D deficiency rickets. J. Steroid Biochem. Mol. Biol..

[9] Lips P. (2007). Relative value of 25(OH)D and 1,25(OH)2D measurements. J. Bone Miner. Res..

[10] Institute of Medicine (2011). Dietary Reference Intakes for Calcium and Vitamin D.

[11] Looker A.C., Johnson C.L., Lachner D.A., Pfeiffer C.M., Schleicher R.L., Sempos C.T. (2011). Vitamin D status: United States, 2001–2006. NCHS Data Brief..

[12] Holick M.F. (2016). Biologic effects of sunlight, ultraviolet radiation, visible light, infrared, and vitamin D for health. Anticancer Res..

[13] Rizzoli R., Biver E., Brennan-Speranza T.C. (2021). Nutritional intake and bone health. Lancet Diabetes Endocrinol..

[14] Navarro D.F., García-Franco A.L., de Guzmán E.N., Rabassa M., Campos R.Z., Pardo-Hernández H., Ricci-Cabello I., Canelo-Aybar C., Meneses-Echavez J.F., Yepes-Nuñez J.J. (2021). Vitamin D recommendations in clinical guidelines: A systematic review, quality evaluation and analysis of potential predictors. Int. J. Clin. Pract..

[15] McKenna M.J. (1992). Differences in vitamin D status between countries in young adults and the elderly. Am. J. Med..

[16] Holick M.F. (1995). Environmental factors that influence the cutaneous production of vitamin D. Am. J. Clin. Nutr..

[17] Takeuchi A., Okano T., Tanda M., Kobayashi T. (1991). Possible origin of extremely high contents of vitamin D3 in some kinds of fish liver. Comp. Biochem. Physiol..

[18] Sunita Rao D., Raghuramulu N. (1996). Food chain as origin of vitamin D in fish. Comp. Biochem. Physiol. A.

[19] Roseland J.M., Phillips K.M., Patterson K.Y., Pehrsson P.R., Taylor C.L. (2018). Vitamin D in foods: An evolution of knowledge. Vitamin D, Volume 2: Health, Disease and Therapeutics.

[20] De Giuseppe R., Tomasinelli C.E., Cena H., Braschi V., Giampieri F., Preatoni G., Centofanti D., Princis M.P., Bartoletti E., Biino G. (2022). Development of a Short Questionnaire for the Screening for Vitamin D Deficiency in Italian Adults: The EVIDENCe-Q Project. Nutrients.

[21] Lips P., Cashman K.D., Lamberg-Allardt C., Bischoff-Ferrari H.A., Obermayer-Pietsch B., Bianchi M.L., Stepan J., El-Hajj Fuleihan G., Bouillon R. (2019). Current vitamin D status in European and Middle East countries and strategies to prevent vitamin D deficiency: A position statement of the European Calcified Tissue Society. Eur. J. Endocrinol..

[22] Musgrave K.O., Giambalvo L., Leclerc H.L., Cook R.A., Rosen C.J. (1989). Validation of a quantitative food frequency questionnaire for rapid assessment of dietary calcium intake. J. Am. Die. Assoc..

[23] Xu L., Porteous J.E., Philips M.R., Zheng S. (2000). Development and validation of a calcium intake questionnaire in postmenopausal women in China. Ann. Epidemiol..

[24] Nelson M., Hague G.F., Cooper C., Bunker V.W. (1988). Calcium intake in the elderly: Validation of a dietary questionnaire. J. Hum. Nutr. Diet..

[25] Taitano R.T., Novotny R., Davis J.W., Ross P.D., Wasnich R.D. (1995). Validity of a food frequency questionnaire for estimating calcium intake among Japanese and white women. J. Am. Diet. Assoc..

[26] U.S. Department of Agriculture’s (USDA’s) FoodData Central Lists the Nutrient Content of Many Foods and Provides a Comprehensive List of Foods Containing Vitamin D Arranged by Nutrient Content. https://ods.od.nih.gov/pubs/usdandb/VitaminD-Content.pdf.

[27] CREA. https://www.crea.gov.it/web/alimenti-e-nutrizione/banche-dati.

[28] Nowson C.A., Margerison C. (2002). Vitamin D intake and vitamin D status of Australians. Med. J. Aust..

[29] Gibson S.A., Ashwell M. (1997). New vitamin D values for meat and their implication for vitamin D intake in British adults. Proc. Nutr. Soc..

[30] Moore C., Murphy M.M., Keast D.R., Holick M.F. (2004). Vitamin D intake in the United States. J. Am. Diet. Assoc..

[31] Herrick K.A., Storandt R.J., Afful J., Pfeiffer C.M., Schleicher R.L., Gahche J.J., Potischman N. (2019). Vitamin D status in the United States, 2011–2014. Am. J. Clin. Nutr..

[32] Ahmed M., Ng A., L’Abbe M.R. (2021). Nutrient intakes of Canadian adults: Results from the Canadian Community Health Survey (CCHS)-2015 public use microdata file. Am. J. Clin. Nutr..

[33] Page P., Steer T., Bates B., Cox L.J., Nicolson S., Prentice A., Swan G. (2016). National Diet and Nutrition Survey Results from Years 5 and 6 (Combined) of the Rolling Programme (2012/2013–2013/2014). A Survey Carried Out on Behalf of Public Health England and the Food Standards Agency. https://www.gov.uk/government/statistics/ndns-results-from-years-5-and-6-combined.

[34] Scientific Advisory Committee on Nutrition (2016). Report on Vitamin D and Health. https://assets.publishing.service.gov.uk/government/uploads/system/uploads/attachment_data/file/537616/SACN_Vitamin_D_and_Health_report.pdf.

[35] Cashman K.D. (2022). Global differences in vitamin D status and dietary intake: A review of the data. Endocr. Connect..

[36] Rippin H.L., Hutchinson J., Evans C.E.L., Jewell J., Breda J.J., Cade J.E. (2018). National nutrition surveys in Europe: A review on the current status in the 53 countries of the WHO European region. Food Nutr. Res..

[37] Turrini A., Saba A., Perrone D., Cialfa E., D’Amicis A. (2001). Food consumption patterns in Italy: The INN-CA Study 1994–1996. Eur. J. Clin. Nutr..

[38] Scalfi L., Brighenti F., Bordoni A., Biagi P., Rossi L., Maiani G., Avigliano L., Strazzullo P., Rotilio G., Rossi L. (2014). LARN, Livelli di Assunzione di Riferimento di Nutrienti ed Energia per la Popolazione Italiana.

[39] Leclercq C., Arcella D., Piccinelli R., Sette S., Le Donne C., Turrini A., On Behalf of the INRAN-SCAI 2005-06 Study Group (2009). The Italian National Food Consumption Survey INRAN-SCAI 2005-06: Main results in terms of food consumption. Public Health Nutr..

[40] Leclercq C., Piccinelli R., Arcella D., Le Donne C. (2004). Food consumption and nutrient intake in a sample of Italian secondary school students. Results from the INRANRM-2001 food survey. Int. J. Food Sci. Nutr..

[41] Rendina D., De Filippo G., Merlotti D., Di Stefano M., Succoio M., Muggianu S.M., Bianciardi S., D’Elia L., Coppo E., Faraonio R. (2019). Vitamin D Status in Paget Disease of Bone Efficacy-Safety Profile of Cholecalciferol Treatment in Pagetic Patients with Hypovitaminosis D. Calcif. Tissue Int..

[42] Cesareo R., Attanasio R., Caputo M., Castello R., Chiodini I., Falchetti A., Guglielmi R., Papini E., Santonati A., Scillitani A. (2018). Italian Association of Clinical Endocrinologists (AME) and Italian Chapter of the American Association of Clinical Endocrinologists (AACE) Position Statement: Clinical management of Vitamin D deficiency in adults. Nutrients.

[43] Bertoldo F., Cianferotti L., Di Monaco M., Falchetti A., Fassio A., Gatti D., Gennari L., Giannini S., Girasole G., Gonnelli S. (2022). Definition, Assessment, and Management of Vitamin D Inadequacy: Suggestions, Recommendations, and Warnings from the Italian Society for Osteoporosis, Mineral Metabolism and Bone Diseases (SIOMMMS). Nutrients.

[44] Foroni M.Z., Cendoroglo M.S., Sakane E.N., Marin-Mio R.V., Moreira P.F.D.P., Maeda S.S., Lazaretti-Castro M. (2023). Serum 25 hydroxyvitamin D concentrations in individuals over 80 years old and their correlations with musculoskeletal and health parameters. Endocrine.

[45] Adami S., Giannini S., Bianchi G., Sinigaglia L., Di Munno O., Fiore C.E., Minisola S., Rossini M. (2009). Vitamin D status and response to treatment in post-menopausal osteoporosis. Osteoporos. Int..

[46] Song Y., Wang L., Pittas A.G., Del Gobbo L.C., Zhang C., Manson J.E., Hu F.B. (2013). Blood 25-hydroxy vitamin D levels and incident type 2 diabetes: A meta-analysis of prospective studies. Diabetes Care..

[47] Autier P., Boniol M., Pizot C., Mullie P. (2014). Vitamin D status and ill health: A systematic review. Lancet Diab. Endocrinol..

[48] Pittas A.G., Kawahara T., Jorde R., Dawson-Hughes B., Vickery E.M., Angellotti E., Nelson J., Trikalinos T.A., Balk E.M. (2023). Vitamin D and Risk for Type 2 Diabetes in People With Prediabetes: A Systematic Review and Meta-analysis of Individual Participant Data From 3 Randomized Clinical Trials. Ann. Intern. Med..

[49] Bouillon R., Manousaki D., Rosen C., Trajanoska K., Rivadeneira F., Richards J.B. (2022). The health effects of vitamin D supplementation: Evidence from human studies. Nat. Rev. Endocrinol..

[50] Kouvari M., Panagiotakos D.B., Chrysohoou C., Yannakoulia M., Georgousopoulou E.N., Tousoulis D., Pitsavos C. (2020). ATTICA study Investigators. Dietary vitamin D intake, cardiovascular disease and cardiometabolic risk factors: A sex-based analysis from the ATTICA cohort study. J. Hum. Nutr. Diet..

[51] Wang R., Wang W., Hu P., Zhang R., Dong X., Zhang D. (2021). Association of Dietary Vitamin D Intake, Serum 25(OH)D3, 25(OH)D2 with Cognitive Performance in the Elderly. Nutrients.

[52] Wilson L.R., Tripkovic L., Hart K.H., Lanham-New S.A. (2017). Vitamin D deficiency as a public health issue: Using vitamin D2 or vitamin D3 in future fortification strategies. Proc. Nutr. Soc..

[53] Lamberg-Allardt C. (2006). Vitamin D in foods and as supplements. Biophys. Mol. Biol..

[54] Chevalley T., Brandi M.L., Cashman K.D., Cavalier E., Harvey N.C., Maggi S., Cooper C., Al-Daghri N., Bock O., Bruyère O. (2022). Role of vitamin D supplementation in the management of musculoskeletal diseases: Update from an European Society of Clinical and Economical Aspects of Osteoporosis, Osteoarthritis and Musculoskeletal Diseases (ESCEO) working group. Aging Clin. Exp. Res..

[55] Liu J. (2012). Vitamin D content of food and its contribution to vitamin D status: A brief overview and Australian focus. Photochem. Photobiol. Sci..

[56] Holden J.M., Lemar L.E. (2008). Assessing vitamin D contents in foods and supplements: Challenges and needs. Am. J. Clin. Nutr..

[57] Willett W.C. (1998). Nutritional Epidemiology.

